# Supplementary material for: Nicotinamide adenine dinucleotide is transported into mammalian mitochondria
Source: eLife. 2018 Jun 12;7:e33246. doi: 10.7554/eLife.33246 (PMC6013257; doi:10.7554/eLife.33246)
Supplement: Supplementary file 1. — Two independent guides were used to target each NMNAT isoform, and were compared to a control targeting the ROSA26 locus. [file elife-33246-supp1.doc]

Supplementary File 1. Table of gRNA sequences cloned into LentiCRISPR v2 vector backbone.

| **CRISPR target** | **name** | **Sequence- FOR 5’--> 3’** | **REV 5’--> 3’** |
| --- | --- | --- | --- |
| CRISPR Ctrl (ROSA26) | R26 | CACCGAAGATGGGCGGGAGTCTTCT | AAACAGAAGACTCCCGCCCATCTTC |
| NMNAT 1 – gRNA 1 | 1a | CACCGTTCTTGTACGCATCACCGAC | AAACGTCGGTGATGCGTACAAGAAC |
| NMNAT 1 – gRNA 2 | 1b | CACCGGTTCTGCCATGATGATTCGG | AAACCCGAATCATCATGGCAGAACC |
| NMNAT 2 – gRNA 1 | 2a | CACCGGCAGGCCAGCAGGATAACGT | AAACACGTTATCCTGCTGGCCTGCC |
| NMNAT 2 – gRNA 2 | 2b | CACCGTCCAGAATTCCGACTGGATC | AAACGATCCAGTCGGAATTCTGGAC |
| NMNAT 3 – gRNA 1 | 3a | CACCGCGCAGGTGCATATTCGTGAT | AAACATCACGAATATGCACCTGCGC |
| NMNAT 3 – gRNA 2 | L3b | CACCGGCCATGGCCACTCGGTGAT | AAACATCACCGAGTGGCCATGGCC |
