# Supplementary material for: Nicotinamide adenine dinucleotide is transported into mammalian mitochondria
Source: eLife. 2018 Jun 12;7:e33246. doi: 10.7554/eLife.33246 (PMC6013257; doi:10.7554/eLife.33246)
Supplement: Supplementary file 2. — Each primer pair amplifies a region just downstream of the guide RNA that bears the corresponding name (see Supplementary file 1). [file elife-33246-supp2.doc]

Supplementary File 2. Primer sequences used to detect NMNAT transcripts.

| **RT-PCR Gene target** | **name** | **Sequence- FOR 5’--> 3’** | **REV 5’--> 3’** |
| --- | --- | --- | --- |
| 36B4 | 36B4 | TGGGAATTTTGGTGTTTCAGACT | ACCGCATCGTTAGAACCAGAC |
| NMNAT 1a | 1a | CCATCACCAACATGCACCTC | CTTCTTGTAATCACCGA |
| NMNAT 1b | 1b | GCTGGCCAAGGACTATATGC | AAGTTCTGCCATGATGATTC |
| NMNAT 2a | 2a | AGACCACAAAGACCCACGTT | GAGACAATCCCGCCAATCAC |
| NMNAT 2b | 2b | GCACCGTCTCATCATGTGTC | CCATGGGTCCACCCTGATC |
| NMNAT 3a | 3a | TGGTTCCTTCAACCCCATCA | CCACCAGGTCTTTCTTCCCA |
| NMNAT 3b | 3b | GACCTGGTGGCTTCCCATCA | CTCAGCACCTTCACCGTTTC |
