# Supplementary material for: Nicotinamide adenine dinucleotide is transported into mammalian mitochondria
Source: eLife. 2018 Jun 12;7:e33246. doi: 10.7554/eLife.33246 (PMC6013257; doi:10.7554/eLife.33246)
Supplement: Transparent reporting form [file elife-33246-transrepform.doc]

***eLife’s* transparent reporting form**

We encourage authors to provide detailed information *within their submission* to facilitate the interpretation and replication of experiments. Authors can upload supporting documentation to indicate the use of appropriate reporting guidelines for health-related research (see [EQUATOR Network](http://www.equator-network.org/ )), life science research (see the [BioSharing Information Resource](https://biosharing.org/)), or the [ARRIVE guidelines](http://www.plosbiology.org/article/info:doi/10.1371/journal.pbio.1000412) for reporting work involving animal research. Where applicable, authors should refer to any relevant reporting standards documents in this form.

If you have any questions, please consult our Journal Policies and/or contact us: [editorial@elifesciences.org](mailto:editorial@elifesciences.org).

**Sample-size estimation**

- You should state whether an appropriate sample size was computed when the study was being designed
- You should state the statistical method of sample size computation and any required assumptions
- If no explicit power analysis was used, you should describe how you decided what sample (replicate) size (number) to use

Please outline where this information can be found within the submission (e.g., sections or figure legends), or explain why this information doesn’t apply to your submission:

No formal power analyses were used to design experiments. Sample sizes were chosen based on prior experience with similar assays. This information is stated in the Experimental Procedures section under the “Statistics” heading.

**Replicates**

- You should report how often each experiment was performed
- You should include a definition of biological versus technical replication
- The data obtained should be provided and sufficient information should be provided to indicate the number of independent biological and/or technical replicates
- If you encountered any outliers, you should describe how these were handled
- Criteria for exclusion/inclusion of data should be clearly stated
- High-throughput sequence data should be uploaded before submission, with a private link for reviewers provided (these are available from both GEO and ArrayExpress)

Please outline where this information can be found within the submission (e.g., sections or figure legends), or explain why this information doesn’t apply to your submission:

Biological replicates are individually treated samples (cells or isolated mitochondria) whereas technical replicates are repeat assays of the same biological replicate. No outliers were excluded from the presented data. This information is stated in the Experimental Procedures section under the “Statistics” heading.

**Statistical reporting**

- Statistical analysis methods should be described and justified
- Raw data should be presented in figures whenever informative to do so (typically when N per group is less than 10)
- For each experiment, you should identify the statistical tests used, exact values of N, definitions of center, methods of multiple test correction, and dispersion and precision measures (e.g., mean, median, SD, SEM, confidence intervals; and, for the major substantive results, a measure of effect size (e.g., Pearson's r, Cohen's d)
- Report exact p-values wherever possible alongside the summary statistics and 95% confidence intervals. These should be reported for all key questions and not only when the p-value is less than 0.05.

Please outline where this information can be found within the submission (e.g., sections or figure legends), or explain why this information doesn’t apply to your submission:

Data are presented as means +/- SEM and unpaired Student’s t-tests were performed to establish the reported p-values. This information, including N, is provided in each of the figure legends.

(For large datasets, or papers with a very large number of statistical tests, you may upload a single table file with tests, Ns, etc., with reference to sections in the manuscript.)

**Group allocation**

- Indicate how samples were allocated into experimental groups (in the case of clinical studies, please specify allocation to treatment method); if randomization was used, please also state if restricted randomization was applied
- Indicate if masking was used during group allocation, data collection and/or data analysis

Please outline where this information can be found within the submission (e.g., sections or figure legends), or explain why this information doesn’t apply to your submission:

In each experiment on isolated mitochondria, a single isolation was performed and the mitochondria subdivided into the indicated treatment groups. For experiments in unmodified cells, all experimental dishes were split from the same parental dish prior to the experiment. In the case of genetically modified cell lines, all of the cells to be compared were generated simultaneously from the same parent line, and were handled equivalently throughout the study. This information can be found in Experimental Procedures under the “Cell culture and isotopic labeling” heading.

**Additional data files (“source data”)**

- We encourage you to upload relevant additional data files, such as numerical data that are represented as a graph in a figure, or as a summary table
- Where provided, these should be in the most useful format, and they can be uploaded as “Source data” files linked to a main figure or table
- Include model definition files including the full list of parameters used
- Include code used for data analysis (e.g., R, MatLab)
- Avoid stating that data files are “available upon request”

Please indicate the figures or tables for which source data files have been provided:

Source data uploaded to Dryad (doi:10.5061/dryad.qt58k):

Figure 1: a,b,c,d,e,f

Figure 2: a,b,c

Figure 3: a,b,c,d

Figure 4: b,c

Figure 5: a,b,c,d,e,f

Figure 6: a,b,c,d,e,f,g,h

Figure 7: a,b,c,d

Figure 5 – figure supplement 1

Figure 6 – figure supplement 1

Figure 6 – figure supplement 2

Figure 6 – figure supplement 3

Figure 6 – figure supplement 4Figure 6 – figure supplement 5
